# Versatile Features of an Antibody Mimetic Peptide and Its Variants

**DOI:** 10.1002/psc.70005

**Published:** 2025-02-17

**Authors:** Simon Dolles, Simon Leukel, Sabrina Gensberger‐Reigl, Anette Rohrhofer, Lena Rauch‐Wirth, Kübra Kaygisiz, Christopher V. Synatschke, Jan Münch, Barbara Schmidt, Monika Pischetsrieder, Jutta Eichler

**Affiliations:** ^1^ Department of Chemistry and Pharmacy, Medicinal Chemistry, FAU NeW ‐ Research Center New Bioactive Compounds Friedrich‐Alexander‐Universität Erlangen‐Nürnberg Erlangen Germany; ^2^ Department of Chemistry and Pharmacy, Food Chemistry, FAU NeW ‐ Research Center New Bioactive Compounds Friedrich‐Alexander‐Universität Erlangen‐Nürnberg Erlangen Germany; ^3^ Institute of Clinical Microbiology and Hygiene University of Regensburg Regensburg Germany; ^4^ Institute of Molecular Virology Ulm University Medical Center Ulm Germany; ^5^ Max Planck Institute for Polymer Research Mainz Germany

**Keywords:** antibody mimetic peptides, D‐amino acid peptides, gp120, HIV‐1, infection enhancement, mAb b12, peptide nanofibrils

## Abstract

Antibody mimetic peptides have evolved as versatile tools for biomedical applications, based on their ability to interfere with protein–protein interactions. We had previously designed a functional mimic of the broadly neutralizing HIV‐1 antibody b12 that recognizes the CD4 binding site of the HIV‐1 envelope glycoprotein gp120. The molecular details of the interaction of a linear variant of this peptide (H1H3s) with gp120 have now been characterized through cross‐linking mass spectrometry, confirming the proposed involvement of the CD4 binding site of gp120 in the interaction. In addition, a variant of the b12 mimetic peptide composed mostly of D‐amino acids was shown to be stable towards proteolytic degradation, while the binding and HIV‐1 neutralizing properties were largely preserved. Furthermore, a peptide variant in which aspartate residues were replaced with lysine was shown to strongly enhance infection of cells with HIV‐1 and GALV glycoprotein pseudotyped viral vectors, respectively, introducing this peptide as a tool to facilitate retroviral gene transfer. Collectively, the presented results highlight the versatile potential therapeutic and gene transfer applications of H1H3s and its variants in particular, as well as antibody mimetic peptides in general.

## Graphical abstract

Interaction analysis of the antibody mimetic peptide H1H3s with its target protein through cross‐linking mass spectrometry confirmed the proposed binding site. Binding and virus neutralizing properties were preserved in a proteolytically stable D‐amino acid peptide variant. K➔D exchanges yielded a peptide that assembles into nanofibrils and enhances retroviral infection.

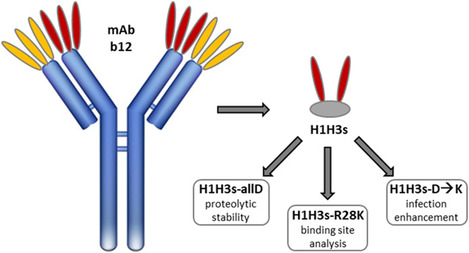

## 1 Introduction

**FIGURE 1:**
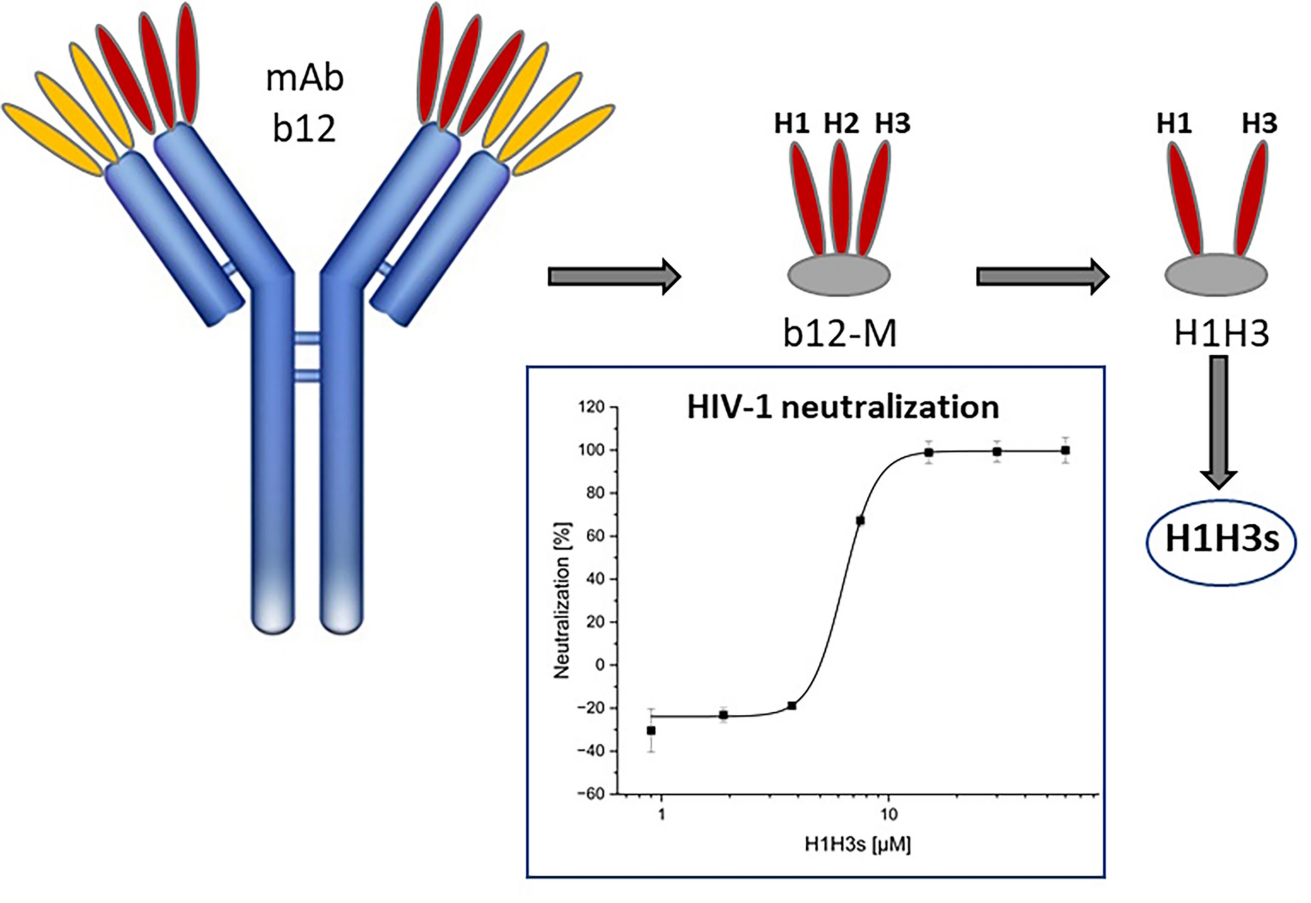
Illustration of the design (top) and HIV‐1 neutralizing activity (bottom) of the b12 mimetic peptide H1H3s. See Materials and Methods section for details.

Over the past two decades, therapeutic antibodies have evolved as one of the most successful class of drugs. The large molecular size of antibodies, however, which may hamper solubility, tissue penetration, as well as access to sterically shielded epitopes, has inspired efforts to design smaller proteins having antibody‐like properties [1]. Furthermore, recombinantly produced antibodies often exist in multiple proteoforms, which may hamper clinical approval due to the challenges of ensuring consistent efficacy, stability, and safety across these variants [2]. Synthetic peptides presenting the complementarity determining regions (CDRs) through which antibodies contact their antigens can be expected to mimic the binding specificities of the antibody [3]. We have previously designed antibody mimetic peptides of mAb b12, which belongs to a group of broadly neutralizing antibodies that recognize the CD4 binding site of HIV‐1 envelope protein gp120 [4]. This peptide (H1H3), which presents the heavy chain CDRs H1 and H3 of b12 (Figure 1), was found to specifically interact with gp120, as well as neutralize HIV‐1, in a b12‐related fashion [5]. In this study, H1H3 served as a starting point for a detailed characterization and optimization with the aim to better understand its mechanism of action, to stabilize it against proteolytic degradation, as well as to probe its utility as an enhancer of retroviral gene transfer.

## 2 Materials and Methods

### 2.1 Peptide Synthesis

Peptides (see Table 1 for sequences) were synthesized as C‐terminal amides by Fmoc/t‐Bu‐based solid‐phase synthesis, as previously described [6]. The N‐terminal amino groups were acetylated with a mixture of acetic anhydride/pyridine/DMF (1:2:3) for 30 min. The side chains of the lysine residue between the H1 and H3 sequence were protected with Alloc and selectively deprotected by treating the peptide‐resin overnight with a solution of Pd (PPh_3_)_4_, (8.6 mg/mL) and 1,3‐dimethylbarbituric acid (13 mg/mL) in DMF under argon. The deprotected amino group was biotinylated using 3 eq. biotin, DIC and oxyma for 4 h. Peptides were cleaved from the resin using Reagent K (TFA/water/phenol/thioanisole/1,2‐ethanedithiol; 82.5:5:5:2.5), precipitated in a cold 1:1 mixture of cyclohexane and *tert*‐butyl methyl ether, extracted with water, and lyophilized. Peptides were purified by preparative HPLC (column: Phenomenex Kinetex C18, 100 × 21.2 mm, flow rate: 30 mL/min, gradient: 20–50% acetonitrile in H_2_O (both containing 0.1% TFA) in 8 min and UV detection at 220 und 280 nm). Peptides were characterized by analytical HPLC with online ESI mass spectrometry detection (LC–MS) (column: Phenomenex Kinetex 2.6 μM C18 100 Å, 50 × 2.1 mm, flow rate: 0.4 mL/min, gradient: 5–95% acetonitrile in H_2_O (both containing 0.1% TFA) in 15 min). Analytical data (LC–MS) of all peptides are given in the Supporting Information. Stock solutions of purified peptides were prepared at 10 mM in DMSO.

### 2.2 gp120 Binding Assay (Figures 2C, 3A, and 4B)

Costar half area microtiter plates were coated overnight at 4°C with gp120 (strain HxBc2, Immune Technology, 1.4 μg/mL) in 0.1 M phosphate buffer pH 7.4 (50 μL per well). Unspecific binding was blocked with Ficoll 400 from Sigma Aldrich (1.0% w/v) in 0.1 M phosphate buffer pH 7.4, 110 μL/well for 1.5 h. Plates were then incubated overnight at 4°C with peptide in serial threefold dilutions, starting at 400 nM. Bound peptides were detected with anti‐Biotin peroxidase conjugate (A4541) from Sigma Aldrich (1:30.000), 50 μL/well for 30 min. Plates were developed with 50 μL/well *o*‐phenylene diamine (OPD) (1 mg/mL) in the presence of 0.03% H_2_O_2_ for approximately 10 min in the dark. After the reaction was stopped with 25 μL/well 2 M H_2_SO_4_, absorbance was read at 492 nm. All data points present means of duplicates.

### 2.3 CD4 Inhibition Assay (Figures 2B and 4C)

Costar half area microtiter plates were coated with gp120 and blocked with Ficoll as described above for the gp120 binding assay. After addition of sCD4 (Sino Biological) at serial threefold dilutions, starting at 72 nM, plates were incubated overnight at 4°C with peptides at 7.5 nM. Bound peptides were detected as described above for the gp120 binding assay.

### 2.4 Generation of Viral Stocks

The plasmid HIV‐1_pBRNL4–3_, kindly provided by F. Kirchhoff, Ulm, was propagated in XL‐1 Blue competent cells (Stratagene – an Agilent Technologies Division, Waldbronn, Germany) and transfected into 293 T cells using FuGENE HD (Roche, Mannheim, Germany). After 48 h, supernatants were cleared by filtering through 0.22 μm Millex‐GS units (Millipore, Schwalbach, Germany) and stored in aliquots at −80°C.

For the generation of GFP‐expressing γ‐retroviral vector pseudotyped with the glycoprotein of gibbon ape leukemia virus (GALV‐RV), 800,000 HEK293T cells were initially seeded and cotransfected as previously described using following plasmids:GFP‐expressing Murine leukemia virus (MLV) vector E200 pcmE26‐gfp (1.15 μg), E848 pCsGPpA‐ed (0.95 μg), and GALV glycoprotein (0.4 μg) [7].

### 2.5 SEAP Reporter Cell HIV‐1 Infection Assay (Figures 1 and 5A)

The indicator cell line CEMx174, which was kindly provided by R. E. Means and R. C. Desrosiers, contained the gene for the secreted alkaline phosphatase (SEAP) under the control of the simian immunodeficiency virus long terminal repeat [8]. Three days after HIV‐1_NL4–3_ infection, cell culture supernatants were removed and analyzed for SEAP activity using the Phospha‐Light kit (Life Technologies, Darmstadt, Germany) according to the manufacturer's recommendations. Data represent mean and standard error of at least three independently performed experiments.

### 2.6 Peptide Digestion with Pancreatic Powder (Figure 4A)

Pancreas powder (from Kreon capsules, Viatris Healthcare GmbH) (6 mg/mL in 0.01 M Tris buffer pH 7.8) was diluted to a final concentration of 0.03 mg/mL. Fmoc‐Trp(Boc)‐OH was added at 50 μM as an internal standard. Peptides were then added to obtain a concentration of 100 μM. Samples of 40 μL of this solution were taken at 0, 1, 3, 9, 27, and 81 min, mixed with 40 μL of a mixture of acetonitrile containing 2% (v/v) TFA to quench the reaction, and analyzed by LC–MS (column: Phenomenex Kinetex 2.6 μM C18 100 Å, 50 × 2.1 mm, flow rate: 0.4 mL/min, gradient: 20%–70% acetonitrile in H_2_O (both containing 0.1% TFA) in 15 min.

### 2.7 Peptide‐gp120 Cross‐Linking and Chymotryptic Digest (Figure 3B)

Approximately 100 μL of a 0.83 μM solution of gp120 HxBc2 (ImmuneTec) was incubated with 0.1 μL of a 8.3 μM peptide solution for 3 h, followed by addition of 0.1 μL of DSBU (disuccinimidyl dibutyric urea from Fisher Scientific, 50 μM in DMSO) for 1 h. The cross‐linking reaction was stopped by addition of 2 μL of 1 mM ammonium bicarbonate. Unreacted peptide, DSBU, and salt were removed by size exclusion chromatography (Zeba® Spin Desalting Columns, 40 kDa MWCO; according to manufacturer's instructions). The resulting solution was lyophilized, dissolved in 100 μL 0.1 M Tris buffer pH 7.8 containing 0.1% (v/v) Protease Max®, and incubated for 20 min at room temperature. Disulfide bridges were reduced by addition of 5 μL 100 mM DTT (dithiothreitol). After 1 h, thiols were alkylated by addition of 7.5 μL 200 mM iodoacetamide for 30 min., followed by quenching of excess of iodoacetamide by adding 10 μL 100 mM DTT. The reduced and alkylated solution was diluted to a total volume of 830 μL with 0.1 M Tris buffer pH 7.8, and chymotrypsin (sequencing grade, from Sigma‐Aldrich) was added at an enzyme/gp120 ratio of 1:25 (m/m). After incubation for 16 h, the reaction was stopped by addition of 10 μL 100% formic acid, followed by lyophilization. The resulting product was dissolved in 100 μL deionized water, purified using Pierce ® C18 tips according to manufacturer's instructions, and lyophilized.

### 2.8 Cross‐Linking Mass Spectrometry (Figure 3C)

The product of the chymotryptic digest of H1H3s‐R28K cross‐linked with gp120 was dissolved in 10 μL 2% acetonitrile/water containing 0.1% formic acid (v/v) and analyzed by reversed‐phase micro liquid chromatography. For this, an Ultimate 3000 RS system consisting of a degasser, pump, autosampler, and column compartment (ThermoFisher, Dreieich, Germany) was coupled to a timsTOF Pro mass spectrometer (Bruker Daltonik, Bremen, Germany) equipped with an electrospray ionization source. The mass spectrometer operated in parallel accumulation serial fragmentation (PASEF) mode with a cycle time of 1.1 s to screen for peptides. A YMC Triart C18 column (500 μm inner diameter, 100 mm length, 1.9 μm particle size; YMC Europe, Dinslaken, Germany) was operated at 35°C with a flow rate of 15 μL/min. Gradient elution was performed with 0.1% formic acid as eluent A and acetonitrile with 0.1% formic acid as eluent B. The eluent composition changed as follows [time (min)/%B]: 0.0/2, 15.0/45, 15.5/80, 20.0/80, 20.1/2, 30.0/2. For instrument control and data acquisition, the otof Control and HyStar software packages (Bruker Daltonik) were utilized. Data evaluation was conducted using DataAnalysis 6.1 (Bruker Daltonik) and MeroX 2.0 software [9, 10]. To enable data processing by MeroX 2.0, the raw data from HyStar/otofControl were converted into the standard mzML format using msConvert. To identify cross‐linked peptides, the following parameters were utilized for MeroX 2.0. Protease cleavage sites were designated at phenylalanine, leucine, tyrosine, and tryptophan, allowing a maximum of three missed cleavages. The peptide length was defined with a minimum of 5 and a maximum of 30 amino acids. Additionally, a focused search for cross‐linked K28 was performed using a minimum length of four amino acids. Carbamidomethylation was applied as a fixed modification, while oxidation on methionine was considered a variable modification. No additional post‐translational modifications were included. Additionally, 6‐aminohexanoic acid, beta‐alanine, and N‐terminal N‐acetyl glycine were introduced as additional amino acids. A maximum cross‐linker distance of 26.9 Å was specified, with specificity 1 at the N‐terminus of K and specificity 2 at the N‐terminus of lysine, serine, threonine, and tyrosine, ensuring consecutive peptides were excluded. A mass error tolerance of 50 ppm for precursor ions and 10 ppm for fragments ions was adopted, with a mass range from 1000 to 6000 Da. Analysis was limited to y‐ and b‐ ions, requiring at least three fragment ions per peptide. Spectra were deisotoped to remove isotopic signals, comparing only potential fragment ions with matching charge states. The quadratic analysis mode was employed to search for cross‐linked peptides. To ensure data reliability, a prescore of 1.0% and a false discovery rate cut‐off of 5.0% were applied. The decoy database was generated by shuffling the amino acid sequences while keeping the protease sites intact.

### 2.9 GALV‐RV Transduction Enhancement Assay (Figure 5C,D)

For the determination of transduction enhancement of GFP‐expressing GALV‐RV, 50,000 Jurkat cells (ATCC, TIB‐152) were seeded in 180 μL RPMI medium (Gibco, 11875093) supplemented with 10% inactivated fetal calf serum (FCS, Gibco, 10437028), 2 mM L‐glutamine (PAN‐Biotech, P04–80050), 100 U/mL penicillin, and 100 μg/mL streptomycin (PAN‐Biotech, P06–07050). Different concentrations of peptides were 1:1 (v/v) mixed with GALV‐RV (1280 dilution on cells) and incubated for 10 min. Subsequently, 20 μL of the virus‐peptide mix was added to cells, and 2 days posttransduction, GFP‐expression was analyzed by flow cytometry using CytoFlex (Beckmann Coulter), and the evaluation was carried out with FlowJo™ (Version 10.8.1).

### 2.10 Transmission Electron Microscopy (Figure 5B)

The peptide was dissolved in DMSO (10 mM stock solution) and then diluted to 10 μM with PBS. Approximately 5 μL of peptide solution was placed on copper grids coated with carbon and formvar layer (300 mesh, Plano GmbH). After 10 min of incubation time, the grids were stained with 4% uranyl acetate solution for 2.5 min and washed with water. Measurements were performed on a Jeol 1400 electron microscope with 120 kV acceleration voltage.

### 2.11 Zeta‐Potential

Approximately 60 μL of preformed peptide nanofibrils (1 mg/mL) was diluted to 600 μL in an aqueous solution of 1 mM KCl. The zeta potential was derived from the electrophoretic mobility of the peptides and measured using a Zeta Nanosizer ZS (Malvern Instruments) with 1 mL of disposable folded capillary cells (Zetasizer Nano series, Malvern). Each measurement was performed in triplicates.

## 3 Results and Discussion

**FIGURE 2:**
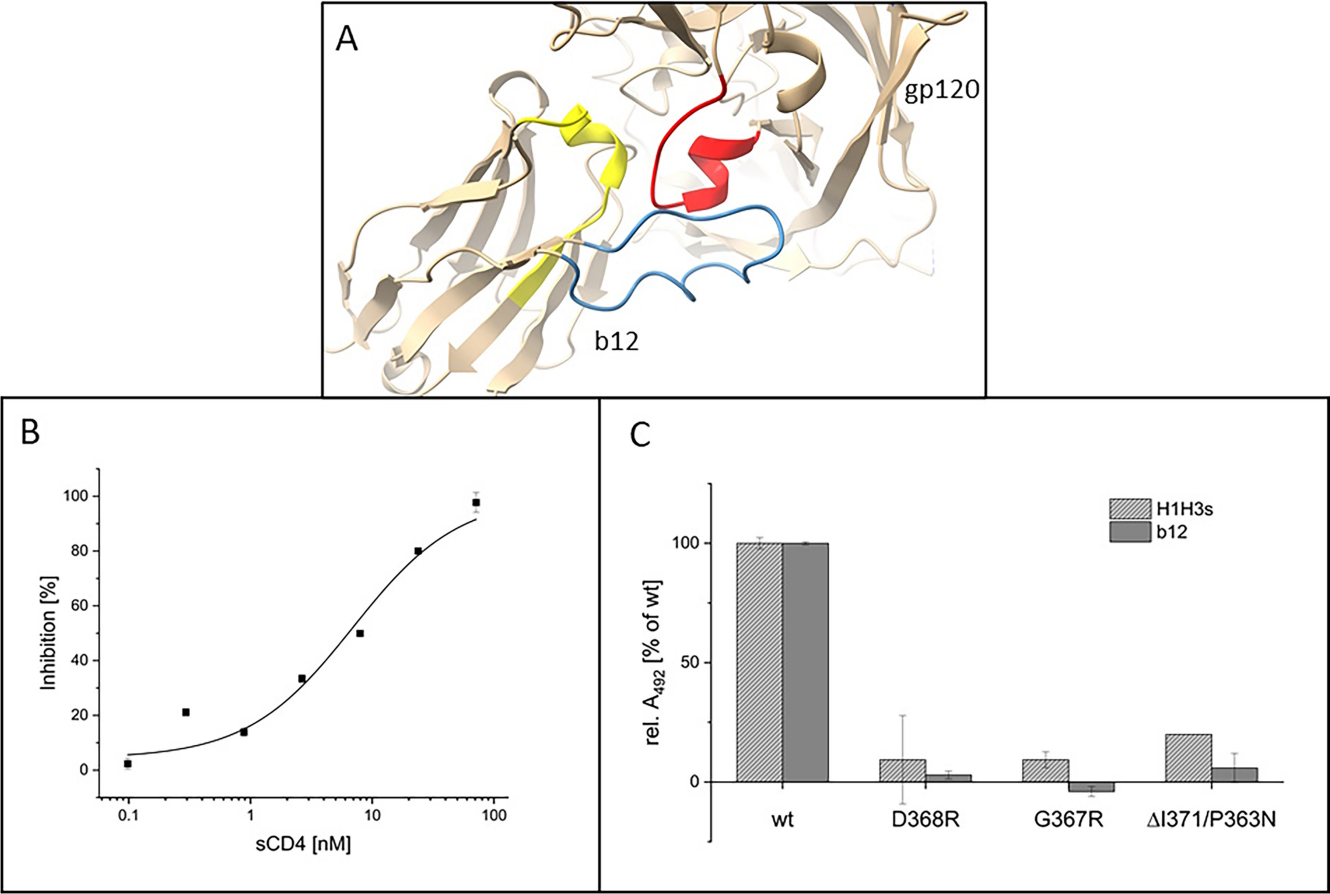
(A) Section of the gp120–b12 complex structure (pdb code 2ny7) depicting the protein interface featuring H1 (positions 26–36, yellow) and H3 (positions 95–101, blue) of b12, as well as the CD4 binding loop of gp120 (positions 365–371, red). (B) Inhibition of the gp120 – H1H3s interaction by sCD4. (C) Interaction of b12 (1.33 nM) and H1H3s (25 nM), respectively, with gp120 variants HxBc2 (wt), AC10.29 D368R, RSC3 G367R and RSC3 ΔI371/P363N. For (B) and (C), average values of three independent experiments are shown. Error bars indicate SD. See Materials and Methods section for details.

The b12 paratope mimetic peptide H1H3 (see Table 1 for peptide sequences), which was designed based on the crystal structure of b12 in complex with gp120 (Figure 2A), presents two of the three CDRs of the antibody's heavy chain, that is, H1 and H3 [11]. Furthermore, the spatial proximity between the N‐ and C‐termini of H3 was covalently stabilized by a disulfide bridge. This was achieved through flanking the H3 sequence by cysteine residues. A lysine residue whose side chain amino group was biotinylated for binding assays was inserted between H1 and H3, along with combinations of the spacer amino acids β‐alanine and ε‐aminohexanoic acid, to provide the required distance between the two antibody fragments (H1 and H3).

**TABLE 1:** Sequences of b12 mimetic peptides.

Peptide	Sequence
H1H3^5^	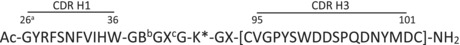
	Ac‐GYRFSNFVIHW‐GB^b^GX^c^G‐K*‐GX‐[CVGPYSWDDSPQDNYMDC]‐NH_2_
H1H3s	Ac‐GYRFSNFVIHW‐GBGXG‐K*‐GX‐SVGPYSWDDSPQDNYMDS‐NH_2_
H1H3s‐allD	Ac‐Gyrfsnfvihw‐GBGXG‐k*‐GX‐svGpyswddspqdnymds‐NH_2_
H1H3s‐L/D	Ac‐GyRfSnFvIhW‐GBGXG‐K*‐GX‐svGpYsWdDsPqDnYmDs‐NH_2_
H1H3s‐D→K	Ac‐GYRFSNFVIHW‐GBGXG‐K*‐GX‐SVGPYSWKKSPQKNYMKS‐NH_2_
H1H3s‐D100^b^K	Ac‐GYRFSNFVIHW‐GBGXG‐K*‐GX‐SVGPYSWDKSPQDNYMDS‐NH_2_
H1H3s‐R28K	Ac‐GYKFSNFVIHW‐GBGXG‐K*‐GX‐SVGPYSWDDSPQDNYMDS‐NH_2_

*Note:* K*, Lys (biotin). Brackets denote disulfide bridges; lower case letters denote D amino acids.

^a^ Position numbers in b12 [6].

^b^ B, β‐alanine.

^c^ X, ε‐aminohexanoic acid.

### 3.1 Characterization of a Linear Variant of the b12 Mimetic Peptide H1H3

Addressing the significance of the disulfide bridge in H1H3, a linear variant (H1H3s) was generated, in which the two cysteine residues were replaced by serine (see Table 1 for peptide sequences). Interestingly, the HIV‐1 neutralizing activity of H1H3 (IC_50_ = 1.7 μM) was largely preserved in the linear variant H1H3s (IC_50_ = 6.3 ± 0.1 μM, Figure 1), suggesting that a covalent stabilization of the loop structure of H3 is beneficial but not essential for interaction with HIV‐1 gp120 [5]. Furthermore, the interaction of H1H3s with HIV‐1 gp120 could be dose‐dependently inhibited by soluble CD4 (sCD4, Figure 2B), indicating an interaction of H1H3s with the CD4 binding site of gp120, which overlaps the epitope of mAb b12 (Figure 2A). This confirms the previously established antibody‐mimetic character of H1H3 also for its linear variant H1H3s [5]. Addressing the binding selectivity of H1H3s, its interaction with wt gp120, as well as with a range of gp120 variants, was compared to b12 (Figure 2C). The tested gp120 variants featured mutations (D368R and G367R/P363N) and deletions (ΔI371), respectively, in the CD4binding site of the protein, which would be expected to interfere with the interaction of the protein with b12. As expected, binding of the gp120 variants to b12 was reduced to below 20% of the interaction with wt gp120. The same was true for the interaction of the gp120 variants with H1H3s, indicating a b12‐like binding specificity of H1H3s.

### 3.2 Exploring the H1H3s–gp120 Interaction Through Cross‐Linking Mass Spectrometry

**FIGURE 3:**
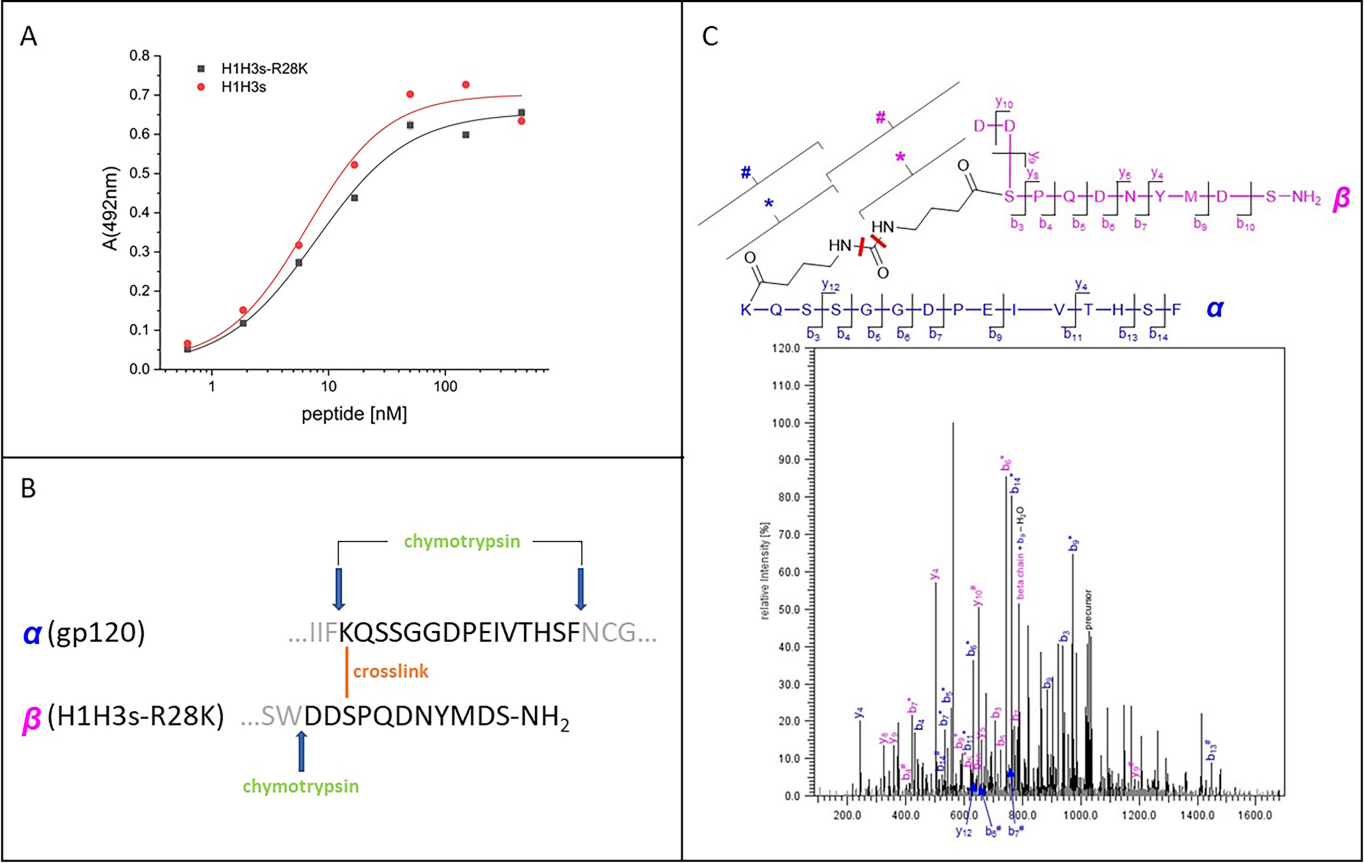
Cross‐linking mass spectrometry. (A) Interaction of H1H3s and H1H3s‐R28K, respectively, with gp120. (B) Fragments of gp120 (α) and H1H3s‐R28K (β), generated through chymotryptic digest of the cross‐linked gp120‐H1H3s‐R28K complex. (C) High‐resolution tandem mass spectrum for the identification of cross‐linked gp120 (α, blue) and H1H3s‐R28K (β, pink). During collision‐induced fragmentation, the cross‐linker is cleaved at different bonds, resulting in fragments denoted by # and *. Key fragment ions that enable unequivocal identification of the cross‐linked peptides are labeled accordingly. For (A), average values of three independent experiments are shown. Error bars indicate SD. See Materials and Methods section for details.

The proposed binding site of H1H3s at or near the CD4 binding site of gp120 was further probed by covalently linking the peptide to gp120, followed by proteolytic digest and mass spectrometric analysis of the resulting peptide fragments [12]. Covalent stabilization of the H1H3s – gp120 interaction was facilitated using DSBU (disuccinimidyl butyric urea) as an MS‐cleavable cross‐linker for amine groups, which yields collision‐induced fragments with a unique mass signature [13]. As the sequence of H1H3s contains no lysine residues, a peptide variant was generated, in which R28 in the H1 fragment was replaced with lysine (H1H3s‐R28K), in order to endow H1H3s with a site for cross‐linking via DSBU. As this modification did not alter the interaction of the peptide with gp120 (EC_50_(H1H3s) = 6.46 ± 1.50 nM; EC_50_(H1H3s‐R28K) = 7.86 ± 1.35 nM, Figure 3A), H1H3s‐R28K was then cross‐linked with gp120, and the resulting covalent complex digested with chymotrypsin. The choice of protease was based on substrate specificity in conjunction with the gp120 fragment of interest. Based on our hypothesis, H1H3s was expected to bind to the CD4 binding site of gp120. The primary component of this binding site, that is, the CD4 binding loop (^365^SGGDPEIVT^373^) of gp120, is flanked by sequences that contain potential cleavage sites for chymotrypsin, that is, ^361^Phe‐Lys^362^ and ^376^Phe‐Asn^377^, whose hydrolysis would yield a fragment presenting the CD4 binding loop, facilitating the identification of cross‐links with the peptide in this region of the protein (Figure 3B). Mass spectrometric analysis of the chymotryptic digest product of H1H3s‐R28K cross‐linked with gp120 identified a cross‐link between K362 of gp120 fragment α (^362^KQSSGGDPEIVTHSF^376^), presenting the CD4 binding loop, and S100^c^ of H1H3s‐R28K fragment β (^100a^DDSPQDNYMDS^101^), presenting part of the H3 sequence of the peptide (Figure 3C).

Interestingly, it was not the additionally introduced lysine residue in the H1 sequence of H1H3‐R28K that was involved in the cross‐link with gp120 but a serine residue within the H3 sequence of the peptide. We specifically searched for potential cross‐links of K28 with adjusted search parameters. However, only one intramolecular cross‐link between K28 and Y98 was identified, and none were detected between gp120 and H1H3. Although this result was initially surprising, it should be noted that DSBU has been shown to cross‐link not only amino groups (lysine) but also hydroxy groups (serine, threonine and tyrosine) [14]. The established cross‐link between K362 of gp120 and S100^c^ of H1H3s‐R28K clearly indicates an interaction of H1H3s‐R28K with the CD4 binding loop of gp120, confirming the b12‐like binding behavior of the antibody mimetic peptide.

### 3.3 A Proteolytically Stable Variant of H1H3s

**FIGURE 4:**
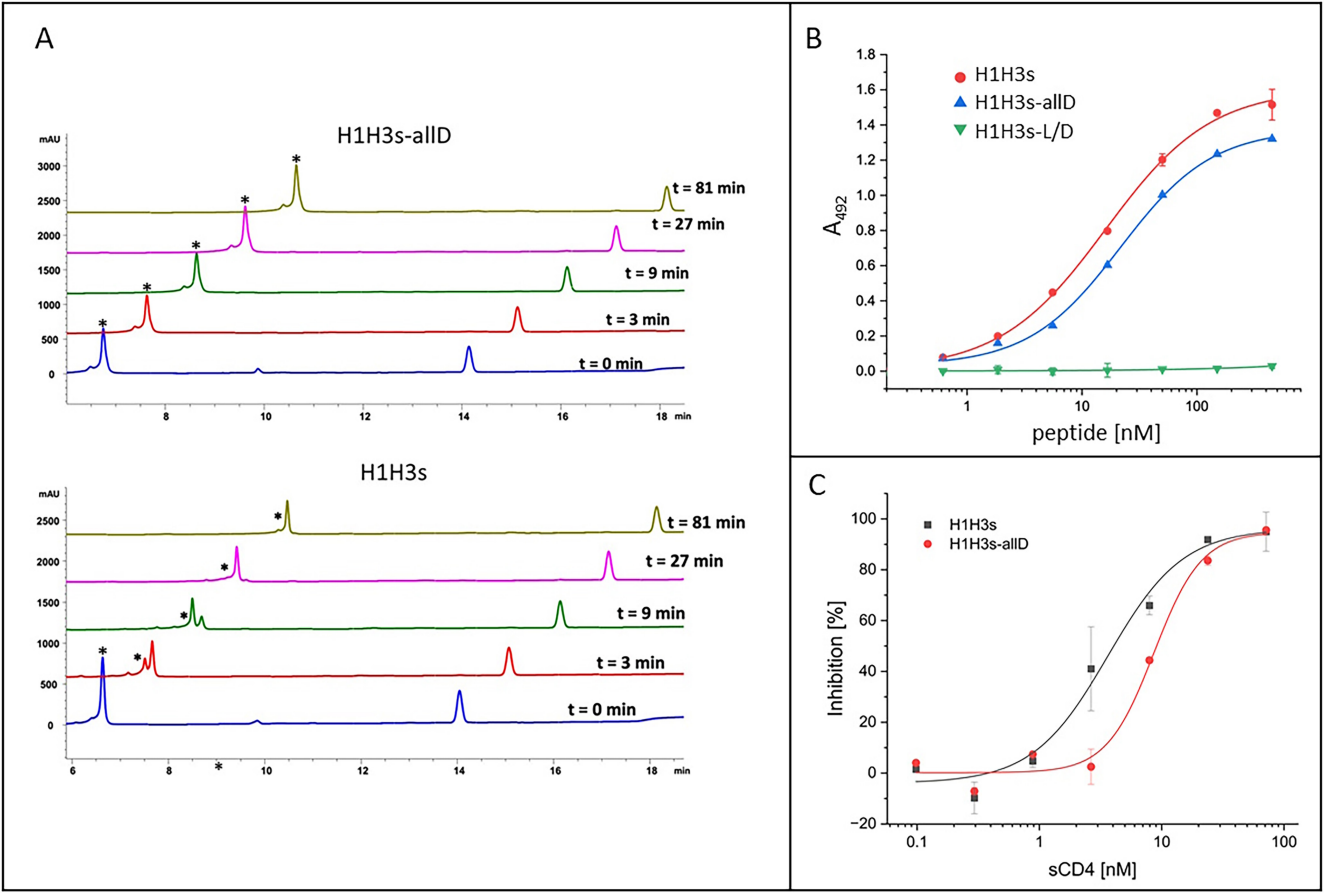
Characterization of H1H3s‐allD compared to H1H3s. (A) HPLC profiles of peptides after incubation with pancreas powder (asterisks denote the original peptides). (B) Dose‐dependent binding of L‐ (H1H3s), D‐ (H1H3s‐allD) and alternating L‐and D‐amino acid (H1H3s‐L/D) peptides to gp120. (C) Inhibition of the peptide‐gp120 interaction by sCD4. C. For (B) and (C), average values of three independent experiments are shown. Error bars indicate SD. See Materials and Methods section for details.

As H1H3s is a flexible, linear peptide composed primarily of proteinogenic amino acids, it would be expected to be prone to proteolytic degradation. Therefore, the susceptibility of H1H3s to proteolytic degradation was probed using a solution of porcine pancreas powder, which contains high amounts of amylases, lipases, and proteases (European Directorate for the Quality of Medicines & HealthCare. 2020; European Pharmacopoeia 11.5.). As expected, H1H3s was rapidly digested by the proteases in pancreas powder (Figure 4A, bottom). In contrast, an H1H3s variant, in which all amino acids of the H1 and H3 sequences had been replaced by the respective D‐amino acids (H1H3s‐allD), was found to be largely stable for more than an hour (Figure 4A, top). While this result was expected, the fact that both the ability of H1H3s to bind to HIV‐1 gp120 (Figure 4B) as well as the inhibition of this interaction by sCD4 (Figure 4C) were preserved in H1H3s‐allD was surprising, as D‐amino exchange acid variants of peptides are generally thought to be active primarily in the shape of retro‐inverso peptides, which present the respective D‐amino acids in reversed sequence [15]. Moreover, the HIV‐1 neutralizing activity of H1H3s‐allD (IC_50_ = 1.9 ± 0.4 μM), which is even slightly higher than that of H1H3s (IC_50_ = 6.3 ± 0.1 μM), confirms the specificity of the effect of H1H3s‐allD, establishing this peptide as a functional H1H3s variant that is largely stable against proteolytic degradation.

While this phenomenon, that is, the essential equipotency of L‐ and D‐amino acid variants of the same peptide sequence, is not common, it has nonetheless been reported for peptides with diverse bioactivities, including antibiotic peptides, as well as peptides interfering with Tau aggregation [16, 17]. These results, however, do not indicate a lack of conformational selectivity of the peptide‐gp120 interaction, as an H1H3s variant, in which the H1 and H3 sequences are presented by alternating L‐ and D‐ amino acids (H1H3s‐L/D), did not bind to gp120 at all (Figure 4B), nor did it inhibit HIV‐1 infection (IC_50_ > 60 μM), pointing to the importance of side‐chain orientation in the peptide. Unlike in peptides composed solely of either L‐ or D‐amino acids, in peptides presenting alternating L‐ and D‐amino acids, all side chains point into the same direction, favoring a curved backbone, which dramatically changes the molecular geometry [18].

### 3.4 An H1H3s Variant as a Tool to Boost Retroviral Gene Transfer

**FIGURE 5:**
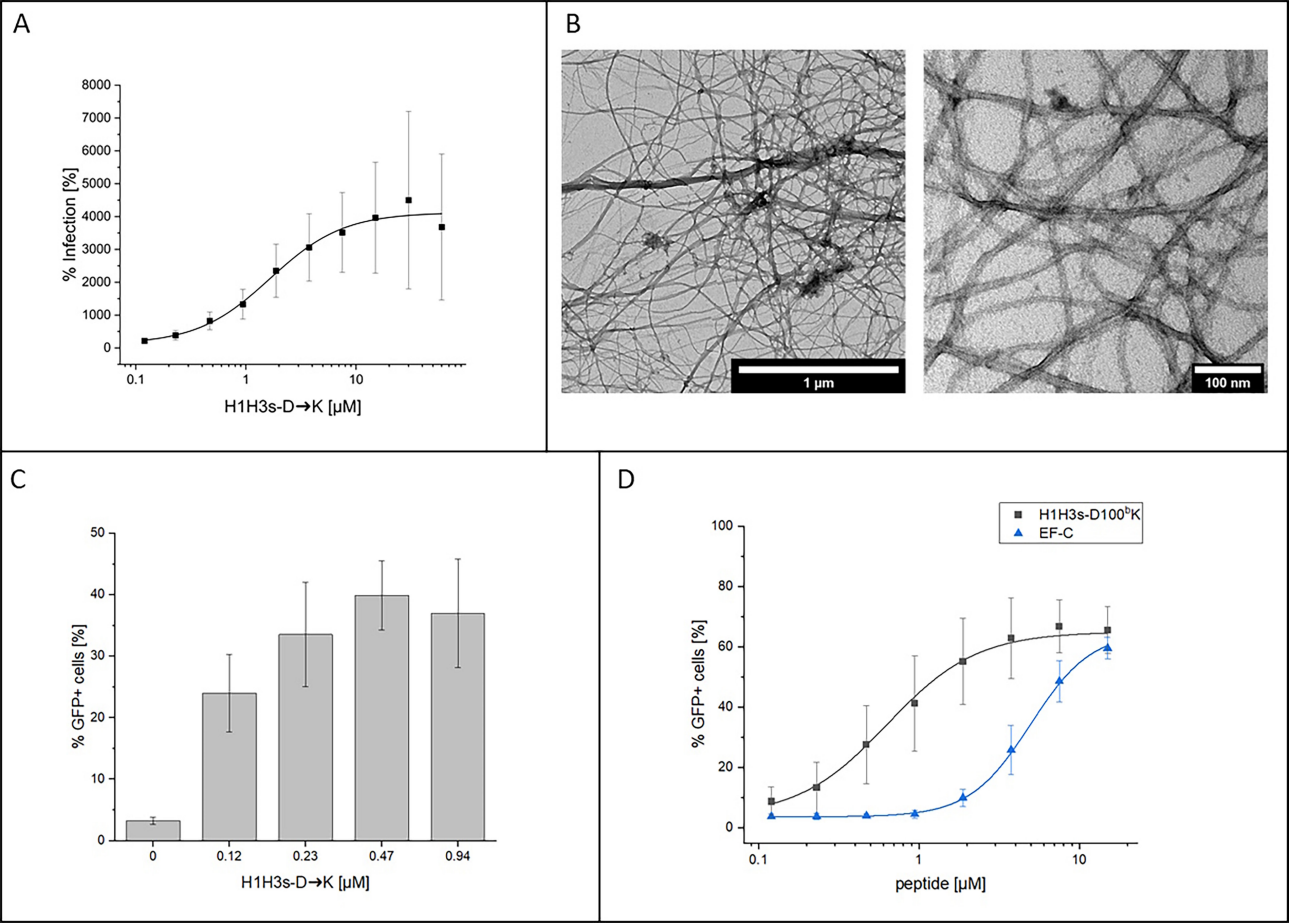
Infection enhancement by D➔K exchange variants of H1H3s. (A) Effect of H1H3s‐D➔K on HIV‐1 infection. (B) H1H3s‐D➔K assembles into nanofibrils. (C) Effect of H1H3s‐D➔K on GALV‐RV transduction. (D) Effect of H1H3s‐D100^b^K and the reference peptide EF‐C^17^ on GALV‐RV transduction. For (A, C and D), average values of three independent experiments are shown. Error bars indicate SD. See Materials and Methods section for experimental details.

As part of the detailed characterization of the binding and virus neutralization properties of H1H3s, a large range of amino acid exchange variants were synthesized and tested, including a peptide, in which all four aspartates were exchanged by lysine residues (H1H3s‐D➔K), dramatically altering the net charge of the peptide from −3 to +5, as well as its zeta‐potential from −4.5 ± 1.1 to +24.3 ± 0.6 mV. Interestingly, rather than inhibiting HIV‐1 infection, H1H3s‐D➔K strongly enhanced it (Figure 5A). A similar effect had previously been observed for a peptide designed to mimic the CD4 binding site of HIV‐1 gp120 [19]. Like this earlier peptide, H1H3s‐D➔K was shown to assemble into nanofibrils (Figure 5B), which mediate the infection enhancing effect, which in turn can be utilized to facilitate retroviral gene transfer [20, 21]. This strategy is widely used for the stable gene delivery into cells, providing substantial benefits for basic research, as well as for therapeutic approaches targeting genetic disorders, cancer, and infections [22]. Despite its advantages, gene transfer applications are often limited by low transduction efficiencies due to insufficient concentrations of infectious virions, as well as by charge repulsion between cellular and viral membranes. These limitations can be amended by peptide nanofibrils that bind to negatively charged viral particles and cellular membranes, enhancing attachment, infection, and transduction rates [23].

The enhancing effect of H1H3s‐D➔K on HIV‐1 infection could also been shown for a GFP‐expressing γ‐retrovirus pseudotyped with the glycoprotein of gibbon ape leukemia virus (GALV‐RV) (Figure 5C), indicating that the infection‐enhancing effect is not specific for HIV‐1. Interestingly, the GALV‐RV‐transduction enhancing effect was even more pronounced in a peptide variant, in which only one of the four aspartates (D100^b^) was replaced with lysine (Figure 5D). The effect of this peptide (H1H3s‐D100^b^K, EC_50_ = 0.63 ± 0.04 μM) was approximately eight‐fold stronger than that of a previously reported peptide booster for retroviral gene transfer (EF‐C, EC_50_ = 4.96 ± 0.14 μM) [24].

## 4 Conclusions

Based on a previously designed functional mimic of the broadly neutralizing HIV‐1 antibody b12 that recognizes the CD4 binding site of the HIV‐1 envelope glycoprotein gp120, we have generated a linear variant of this peptide (H1H3s) and characterized the molecular details of its interaction with gp120 through cross‐linking mass spectrometry, confirming the proposed involvement of the CD4 binding site of gp120 in the interaction. The unexpected preservation of gp120 binding and HIV‐1 neutralizing properties of H1H3s in a peptide variant composed mostly of D‐amino acids exemplifies chemical methods of stabilizing peptides against proteolytic degradation, without losing bioactivity. Furthermore, H1H3s variants in which aspartate residues had been replaced with lysines were introduced as tools to facilitate retroviral gene transfer, as they were shown to strongly enhance infection of cells with HIV‐1 and GALV glycoprotein pseudotyped viral vectors, respectively. In conclusion, the presented results demonstrate the versatile potential features of H1H3s and its variants in particular, as well as antibody mimetic peptides in general. It should be noted that all of the peptide variants generated for this study contained a range of nonproteinogenic amino acids, illustrating the benefits of chemical vs. recombinant peptide and protein synthesis.

## Supporting information


**Figure S1.** HPLC chromatograms and ESI‐mass spectra (insets) of synthesized peptides.

## Data Availability

The data that support the findings of this study are available from the corresponding author upon reasonable request.
