# Supplementary material for: Versatile Features of an Antibody Mimetic Peptide and Its Variants
Source: J Pept Sci. 2025 Feb 17;31(3):e70005. doi: 10.1002/psc.70005 (PMC11833279; doi:10.1002/psc.70005)
Supplement: Supplementary file 1 — Figure S1. HPLC chromatograms and ESI‐mass spectra (insets) of synthesized peptides. [file PSC-31-e70005-s001.pdf]

<u>Supplementary Material</u>

Versatile features of an antibody mimetic peptide and its variants

Simon Dolles[a], Simon Leukel[a], Sabrina Gensberger-Reigl[b], Anette Rohrhofer[c], Lena Rauch-Wirth[d], Kübra Kaygisiz[e], Christopher V. Synatschke[e], Jan Münch[d], , Barbara Schmidt[c] , Monika Pischetsrieder[b] and Jutta Eichler[a]*

[a]Department of Chemistry and Pharmacy, Medicinal Chemistry, FAU NeW - Research Center New Bioactive Compounds, Friedrich-Alexander-Universität Erlangen-Nürnberg, Germany

[b]Department of Chemistry and Pharmacy, Food Chemistry, FAU NeW - Research Center New Bioactive Compounds, Friedrich-Alexander-Universität Erlangen-Nürnberg, Germany

[c]Institute of Clinical Microbiology and Hygiene, University of Regensburg, Germany

[d]Institute of Molecular Virology, Ulm University Medical Center, Ulm, Germany

[e]Max Planck Institute for Polymer Research, Mainz, Germany

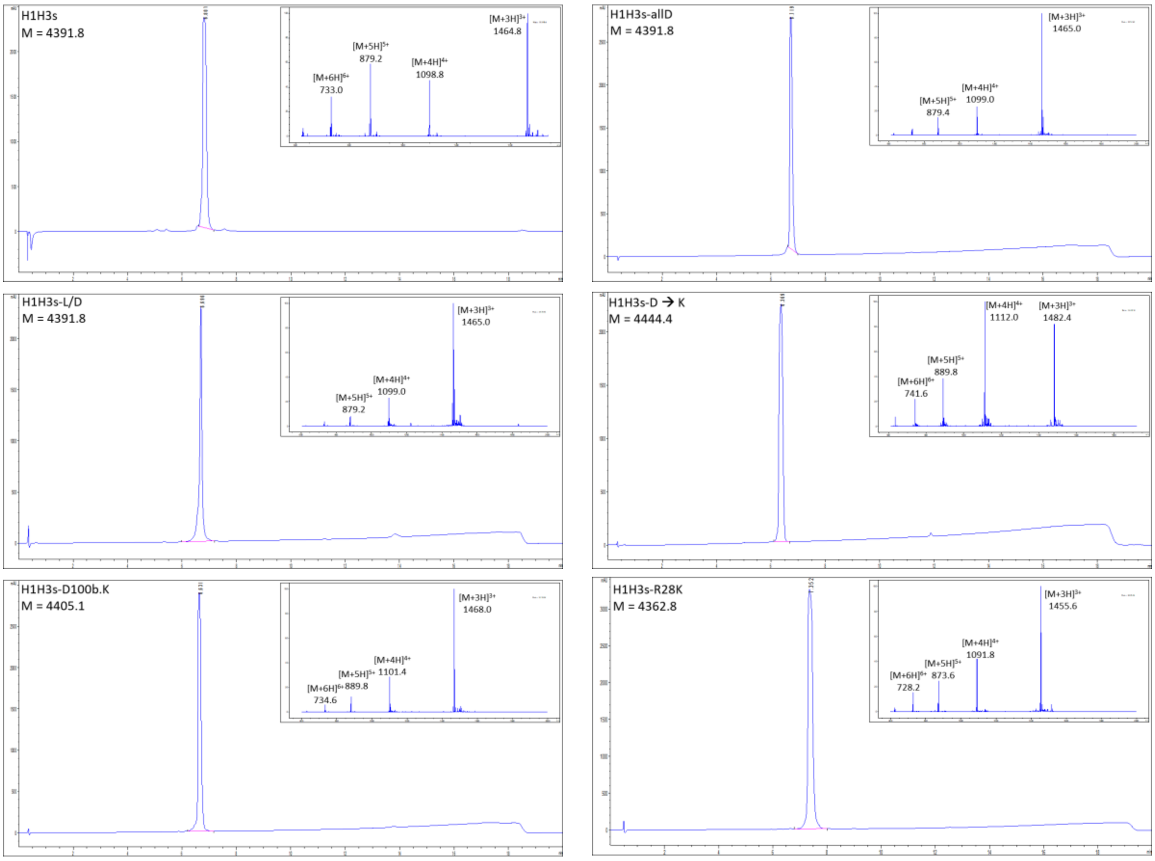

Figure S1: HPLC chromatograms and ESI-mass spectra (insets) of synthesized peptides.
